# Semantic Relation Priming Is Not Constituent-Specific—Evidence from Electrophysiology

**DOI:** 10.3390/brainsci13071033

**Published:** 2023-07-06

**Authors:** Xiaofei Jia, Changle Zhou, Tao Wang

**Affiliations:** 1 Department of Psychology, Qufu Normal University, Qufu 273165, China; jxiaofei2008@126.com; 2 Department of Cognitive Science, Xiamen University, Xiamen 361005, China

**Keywords:** compound word, semantic composition, relation priming, event-related potential

## Abstract

Compound words in psycholinguistics pose a significant challenge for researchers as their meaning involves more than the sum of their parts. The role of semantic relations in this process is crucial, and studies have reported a phenomenon known as relation priming. It suggests that previously encountered relations enhance the processing of subsequent words with the same relation. Notably, this priming effect is limited to cases where there is morpheme repetition between the priming and target words. In the present study, 33 samples from the target group were selected, and the within-subject design of 3 morphemes (modifier-shared, head-shared, non-repeated) × 2 relations (relation-same, relation-different) was adopted to explore whether the relation priming effect would occur without morpheme repetition and its time course. Significant relation priming effects were found in both behavioral and electrophysiological experimental results. These findings indicating relation priming can occur independently of morpheme repetition, and it has been activated at a very early stage (about 200 ms). As the word processing progresses, this activation gradually strengthens, indicating that the relation role is slowly increasing in the process of compound word recognition. It may first be used as context information to help determine the constituent morphemes’ meaning. After the meaning access of the constituent morphemes, they begin to play a role in the semantic composition process. This study uses electrophysiological technology to precisely describe the representation of relation and its time course for the first time, which gives us a deeper understanding of the relation priming process, and at the same time, sheds light on the meaning construction process of compounds.

## 1. Introduction

Concepts are the fundamental building blocks of cognition and other complex mental processes. The process of combining existing concepts to form novel ones provides an effective means to convey fresh meanings. As an illustration, the combination of “computer” and “desk” yields the novel expression “computer desk”. Among various concept combinations, noun-noun combinations are particularly prevalent, with the modifier + head noun subtype garnering considerable research attention [1,2]. The modifier + noun structure falls under the grammatical relationship between the attributive and the head. Semantically, it represents a modifying relationship where the modifier restricts the semantic scope of the head. An illustrative example is the compound word “snowman”, where “snow” serves as a modifier to modify the central noun “man”, resulting in the meaning of “a man made of snow”.

Despite the concise form in which two nouns are combined to create a new concept, the resulting meaning encompasses not only the sum of the constituent concepts but also the semantic relationship between them. For instance, the compound “dog scarf” can be interpreted as “a scarf for a dog to wear” using the “for” relation or as “a scarf with a dog’s image” using the “with” relation [3]. Linguists have endeavored to analyze the various relations employed in forming compound concepts. Building upon this tradition, the CARIN theory posits that compound concepts are constructed by binding two constituents with a specific thematic relation [4]. According to this theory, relations not only serve as the foundation for constructing compound concepts but also play a crucial role in their interpretation. Specifically, interpretation entails the selection of the appropriate relation to depict the connection between the modifier and the head.

The role of the relation in the recognition of compound words has garnered increasing attention from researchers [5,6,7,8]. Relation priming has been observed in various Indo-European languages such as English, Dutch, German, and Italian, as well as Chinese [6,7,8]. For instance, in English, Gagné et al. discovered that the target word in the modifier + head structure could be facilitated by a priming word with the same semantic relation. To illustrate, if participants were previously exposed to “student accusation” (accusation by a student), they would find “student vote” (vote by a student) easier to comprehend compared to “student car” (car of a student) [9,10].

However, there is no consensus on the representation of relations. Gagne et al. (2005) found that the effect of the relationship is bound to morphemes, and no general structural priming was observed. The relation priming effect will occur only when the priming word and the target word share morphemes (or are semantically similar) [11]. But Estes (2006) came to the opposite conclusion. He believes that relation priming is essentially a unique mode of activation; regardless of whether there is morpheme repetition, the facilitated effect of relation will be manifested. Gagne’s experiments failed to find the independent representations of relation, maybe because the semantic classification standards they adopted were rather vague, which did not capture the essence of the relation, so they could not distinguish between the same relation and the different relation well [12]. In Chinese, researchers also found such relation priming [7,8]. Moreover, they found the mode of relation priming is affected by language characteristics and that whether the repeated morpheme is a modifier or head, it can trigger relation priming. However, their experiment only set morpheme repetition conditions, so it is impossible to answer whether relation priming can occur independently of morpheme repetition. Raffray et al. (2007) found that relation priming can occur without morpheme repetition, but morpheme repetition can boost relation priming [3].

Therefore, we infer that relation priming can occur without morpheme repetition. Since the semantic classification used by Gagne et al. is not typical enough, only relatively strong relation priming is found, and the relation priming is much weaker when there is no morpheme repetition, so they failed to detect it.

Chinese is a meaning-spelling language. One person only needs to master seven or eight thousand characters (i.e., semantic bases). The vocabulary formed by combining these bases can meet daily needs [13]. This feature determines that compounding is its primary word-formation method. Moreover, Chinese only has morpheme boundaries, but no word boundaries, so Chinese should have greater freedom of combination and higher productivity to meet their language characteristics. Independent representation of relation seems to be a need. For the above reasons, we use Chinese materials (see the methods section for details). These materials only contain typical instances of each relation (the consistency of the rate between subjects reached above 95%). If the above supposition is correct, then the relation priming should also appear in the absence of morpheme repetition. The facilitative effect under the repeated condition should be higher than that of the non-repeated condition.

In the following experiments, we set up morpheme repetition conditions (modifier or head, corresponding to MS, HS) and morpheme non-repetition conditions (NU). We hypothesize that relation priming will appear under all the conditions. That is, under MS, HS, and NU conditions, the “same relation” will have a greater priming effect than the “different relation”. The relation priming under the morpheme repetition condition is greater than that of the non-repetition condition. That is, the difference between the “same relation” and the “different relation” is more significant under the MS and HS conditions than under the NU condition.

Researchers found that the time when relations begin to take effect is later than when constituent morphemes are semantically activated [8]. Because relations start to work on behalf of the beginning of the meaning composition process, they always occur after the semantic activation of constituent morphemes. But this is limited to the relation associated with the modifier because the relation priming they found occurs under the modifier-repeated condition [8], which is currently the only ERP study on the time course of relation priming. We want to shed light on the relation priming’s time course under head-repeated and no repetition conditions (if there is a relation priming). Therefore, in addition to modifier-repetition conditions, the current research also sets sharing head conditions and non-sharing morpheme conditions (For example, for the target ”书店 (meaning bookstore)”, in addition to “书柜 (meaning bookcase, modifier-repetition condition)”, we also set “饼店 (meaning bakery store, head-repetition condition)” and “菜摊 (meaning vegetable stall, sharing no morpheme condition)”).

This study investigates the pattern of relation priming under shared morpheme condition and non-repetition condition. Some viewpoints hold that the modifier is the first morpheme, which is more critical for whole-word processing than the last morpheme [14]. However, it is generally believed that for the modifier + head structure, the head represents the conceptual category to which the entire word belongs, while the modifier only modifies details, so the head is more critical for whole-word processing [6,15]. In the head-repeated condition, the rough meaning of the whole word would be activated when the head was primed. In this case, would the relation that only serves as a modification still work? If it does, would the pattern be the same as in the modifier-repeated condition? If it was different, what pattern would it show? And if the relation priming exists when there are no repeated morphemes, what pattern would it lead to? Should it be activated at the same time as or after the constituents? If the activation occurs simultaneously with constituents, should it start at the lexical or semantic level? Therefore, besides the N400 time window (relation priming associated with modifier occurs in this window), we would also examine whether the relation activated in an earlier period, such as the morphological processing stage. The central-parietal N200 is an ERP component induced by Chinese two-morpheme compounds recently discovered by Zhang et al. (2012), which appears 200 ms after the stimulus onset, and is centered on the central-parietal area [14]. At present, it is considered a component related to the processing of Chinese orthography. If the relation is activated at this stage, the same relation and different relation conditions should show differences in the N200. Otherwise, there will be no difference.

## 2. Method

### 2.1. Participants

Thirty-two healthy Xiamen University students participated in this experiment voluntarily. All of them were native Chinese speakers, right-handed, aged between 18–25 years (average 21.4 years), and had normal or corrected-to-normal vision. All participants read and signed the written informed consent following a research protocol approved by the IRB board of Xiamen University before the experiment.

### 2.2. Research Design and Materials

We adopted a 3 × 2 within-subjects design. The target includes 72 noun-noun compounds with a “modifier + head” structure (Some of them were selected from [7]). Each target (e.g., 手表 meaning wristwatch) has six primes corresponding to the six conditions: 1. Sharing the modifier and the relation with the target (MSRS, e.g., 手链 meaning hand chain); 2. Sharing the modifier with but differing in the relation from the target (MSRD, e.g., 手背 meaning hand back); 3. Sharing the head and the relation with the target (HSRS, e.g., 怀表 meaning pocket watch); 4. Sharing the head with but differing in the relation from the target (HSRD, e.g., 秒表 meaning stopwatch); 5. Sharing no constituent but having the same relation with the target (NURS, e.g., 颈环 meaning neck ring); 6. Sharing neither constituent nor the relation with the target (NURD, e.g., 丝袜 meaning silk stockings). Please refer to the following table (Table 1) for details.

According to the frequency dictionary of [16], the mean frequency of the target is 0.94 occurrences per million. There was no statistical difference in mean frequency across six conditions (MSRS: 0.91 (sd = 0.34); MSRD: 0.79 (sd = 0.16); HSRS: 1.01 (sd = 0.37); HSRD: 0.89 (sd = 0.19); NURS: 0.85 (sd = 0.17); NURD: 1.03 (sd = 0.26)). Using a 5-point scale (1 means completely unrelated, 2 means unrelated, 3 means unknown, 4 means correlated, and 5 means highly correlated), 15 people from the same subject population rated the prime and target’s semantic relatedness. There was no statistical difference between the RS and RD conditions at the three morpheme levels (MS: 2.47 vs. 2.67, t = −0.42, *p* = 0.68; HS: 3. 00 vs. 2.93, t = 0.19, *p* = 0.85; NU: 2.00 vs. 1.93, t = 0.19, *p* = 0.86). Using a 5-point scale, another 15 people rated the category relatedness between the prime and target, that is, judging the likelihood of the prime and target coming from the same semantic category. And there was no statistical difference between the RS and RD conditions at the three morpheme levels (MS: 2.13 vs. 2.27, t = −0.31, *p* = 0.76; HS: 2.80 vs. 3.07, t = −0.69, *p* = 0.50; NU: 1.80 vs. 1.93, t = −0.44, *p* = 0.67).

One list of 432 filler pairs was also constructed. In half of the word pairs, the prime was interpretable, but the target was nonsense. In the other half, both the prime and the target were nonsense. Matched with the test pairs, in 2/6 (144 pairs) of the total filler pairs, the first morpheme was repeated. In another 2/6 (144 pairs), the second morpheme was repeated, and the remaining 2/6 did not have repeated morphemes. All the test and filler pairs were divided into six parts, forming six versions. Each version contained 72 test pairs and 72 filler pairs. Each target appeared only once in each version, and its corresponding six primes appeared in the six versions, respectively. Each participant used one version for a test.

### 2.3. Procedure

We use the E-prime behavioral response system and adopt a sense-nonsense judgment task and a priming paradigm. The participants sat in a sound-attenuated and dimly lit room facing the screen at a distance of 0.7 m while placing the index fingers of the left and right hands on the F and J keys of the keyboard, respectively. They were asked to look at the fixation in the center of the screen and relax. At the beginning of each trial, “Ready?” appeared in the center of the screen. When ready, they could press the Q key to initiate each trial. First, the prime appeared, and the participant judged whether the word was meaningful or not and responded by pressing the corresponding key (F or J) as quickly as possible. After they responded, the message “Ready?” appeared again, and the participants pressed the Q key to display the target. Likewise, the participants indicated whether the target was meaningful or not by pressing the F or J key. Two adjacent trials were a unit, where the first presented the prime, and the second showed the target, but this pair structure was not made known to them. The self-paced presentation gave the participants enough time to process each word and ensured they could fully understand each word and respond according to its semantic sensibility.

### 2.4. EEG Recording and Data Analysis

During the experimental task, we recorded EEG (Brain cells are conducting spontaneous, rhythmic, and comprehensive electrical activities all the time. Taking the potential of this electrical activity as the vertical axis and time as the horizontal axis, the plane diagram of the relationship between the recorded potential and time is called an electroencephalogram (EEG)) to observe the effect of different priming conditions on the event-related potential evoked by the target. The participants were required to avoid unnecessary head movement and control blinking during the stimulus presentation and responding during recording sessions. Using NEUROSCAN 4.5 with 64-channel Ag/AgCl electrode caps, the electrodes are placed according to the extended 10–20 system, the band-pass filtering range is 0.1–70 Hz, and the sampling rate is 500 Hz. The impedance in all electrodes was less than 7 kΩ. The electrodes for recording the vertical EOG (EOG is a kind of eye electrical signal, which is mainly used to denoise EEG/ERP signal) were located above and below the left eye, and the electrodes for recording the horizontal EOG were located on the left and right lateral orbital rim. Taking the tip of the nose as a physical reference, the original EEG was recorded continuously and was re-referenced offline to the mean of the bilateral mastoids. EEGLAB 14.1.1 was used for data analysis. Epochs were computed from 200ms before to 800 ms after the stimulus onset, and the baseline correction was made between −200 to 0 ms. The offline band-pass filtering was applied between 0.1–30 Hz, and independent component analysis (ICA) was used to remove ocular artifacts. Incorrect responses and responses with amplitude greater than ±75 µv were excluded from the superimposed average. The data rejected due to artifacts is 8.5%.

## 3. Results

### 3.1. Behavioral Results

**Figure 1:**
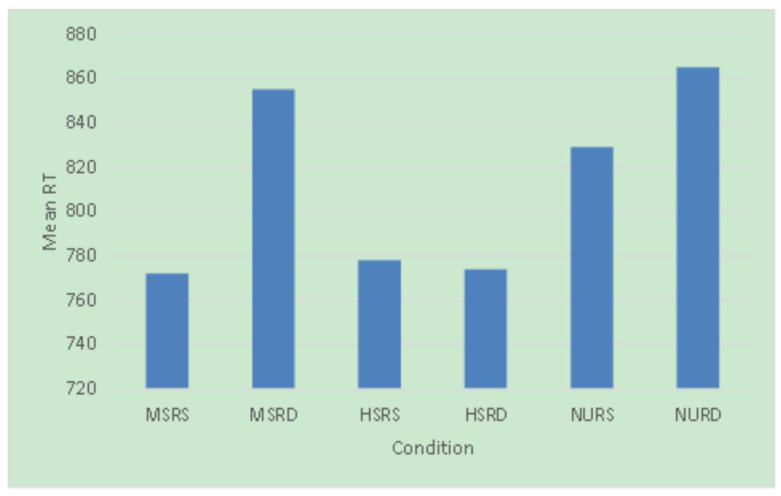
Mean RT for all conditions.

For the response time data (see Figure 1 for details), we used the LmerTest package of R software to fit a mixed-effects model with logarithmic response time as the dependent variable, morphemes (MS, HS, NU) and relations (RS, RD) as fixed factors, and subject and item as random factors [17,18,19]. We found that morphemes have a main effect, F (2, 2287.4) = 6.99, *p* = 0.0009, and the relation also has a main effect, F (1, 2276.2) = 7.19, *p* = 0.0074, but there is no interaction between them, F (2, 2275.6) = 0.97, *p* = 0.3797. Contrast analysis showed that the response time of NU conditions was significantly slower than that of MS and HS (MS vs. NU: 794 vs. 850 ms, β = −77.94, SE = 30.30, t = −2.57, *p* = 0.0102; HS vs. NU: 771 vs. 850 ms, β = −72.58, SE = 30.57, t = −2.37, *p* = 0.0177), while there was no significant difference between MS and HS (MS vs. HS: 794 vs. 771 ms, β = 5.36, SE = 30.31, t = 0.18, *p* = 0.8597). The response time of the RS condition is significantly faster than that of the RD condition (RS vs. RD: β = 80.92, SE = 30.42, t = 2.66, *p* = 0.0079). The logistic regression method was used to analyze the accuracy of data. Because the accuracy reached the ceiling (above 98%), all the analyses were statistically non-significant.

### 3.2. ERP Results

**Figure 2:**
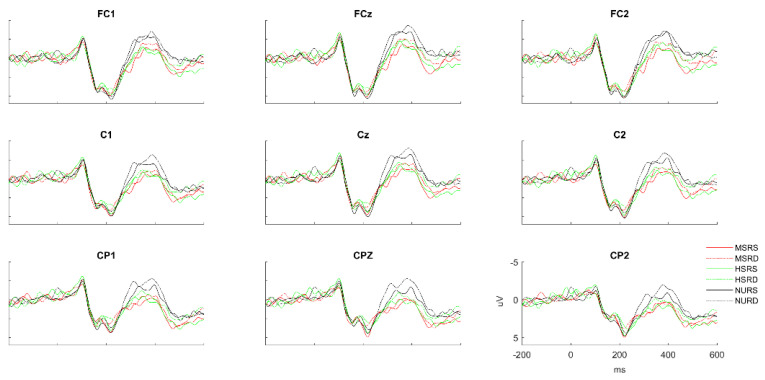
Grand average ERP waveforms for all conditions in 9 representative electrodes. MSRS: prime sharing the modifier and the relation with the target; MSRD: prime sharing the modifier with but differing in the relation from the target; HSRS: prime sharing the head and the relation with the target; HSRD: prime sharing the head with but differing in the relation from the target; NURS: prime sharing no constituent but have the same relation with the target; NURD: prime sharing neither constituent nor the relation with the target.

**Figure 3:**
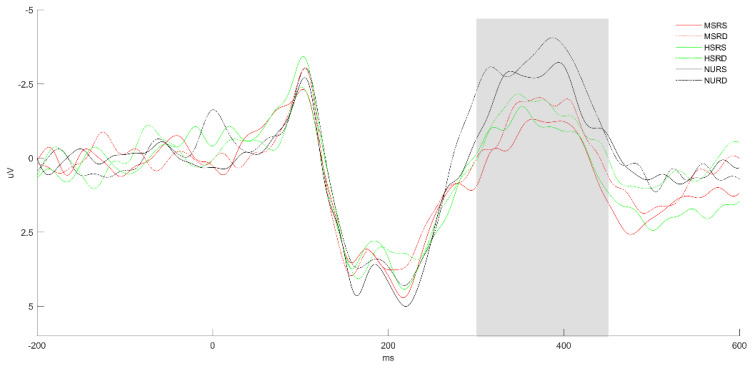
Electrode Cz from Figure 2 highlighted for clarity. Legends are the same as in Figure 2.

**Figure 4:**
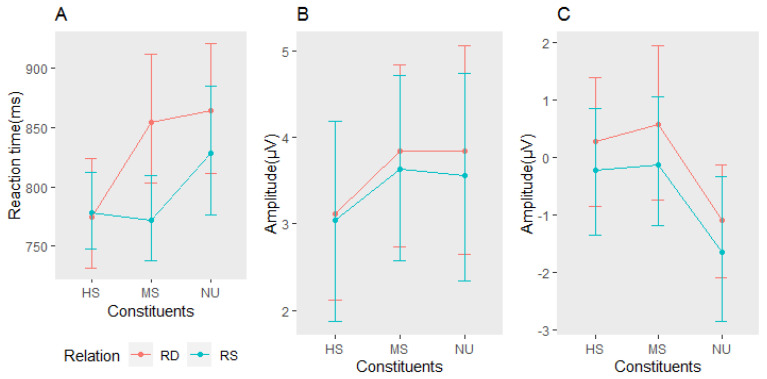
Relation effects on Reaction time (**A**), N200 (**B**), and N400 (**C**). RS: prime sharing the same relation with the target; RD: prime having different relation from the target.

Figure 2 shows the average ERP response on the representative electrodes using different colors for the six conditions (MSRS, MSRD, HSRS, HSRD, NURS, and NURD). Waveforms at electrode Cz are highlighted in Figure 3 for clarity. It can be seen that the above six conditions all elicited a clear N400. We take a 300–450 ms time window and do a morpheme (3) *relation (2) repeated-measures ANOVAs of the averaged amplitude over 9 electrodes (FC1, FCz, FC2; C1, Cz, C2; CP1, CPz, CP2). We found that morphemes have a main effect, F (2, 155) = 11.54, *p* < 0.0001, and the relation also have a main effect, F (1, 155) = 4.01, *p* = 0.047, but there is no interaction between them, F (2, 155) = 0.03, *p* = 0.967. Contrast analysis showed that there is a significant reduction in amplitude of MS and HS relative to NU, and there was no significant difference between them (MS vs. NU: 0.52 vs. −1.07 μV, t (155) = 3.23, *p* = 0.0015; HS vs. NU: 0.32 vs. −1.07 μV, t (155) = 2.67, *p* = 0.0084; MS vs. HS: 0.52 vs. 0.32 μV, t (155) = −0.56, *p* = 0.5734). RS also showed a significant reduction in amplitude compared with RD conditions (RS vs. RD: −1.07 vs. −1.66 μV, t (155) = −2.00, *p* = 0.047). Because the relation information is our focus, to show it more clearly, we offer the relation effects of MS, HS, and NU conditions on behavior, N200, and N400 separately, as shown in Figure 4.

To test whether this relation effect appeared in the earlier period (N200), we took a time window of 185–207 ms and performed a morpheme (3) × relation (2) repeated-measures ANOVAs on the average amplitude of the nine electrodes mentioned above. No main effects were found in the morphemes and relations (F (2, 155) = 1.93, *p* = 0.149; F (1, 155) = 0.38, *p* = 0.537), and there was also no interaction between them (F (2, 155) = 0.04, *p* = 0.960). However, as shown in Figure 4B, although it did not show a significant difference, there is a trend of relation effects. That is, RS conditions have increased amplitude relative to RD in MS and NU, except for HS.

## 4. Discussion

As the behavioral results indicated, MS and HS show better behavioral performance than NU because there is a repetition of the first morpheme between the prime and target, reflecting the repetitive priming effect of the morpheme and the semantic priming at the whole word level. Under all these three conditions, RS has better behavior performance than RD because there is a repetition of the relation between the prime and target, reflecting the effect of relation priming. It’s just that the relation priming effect under the constituent-repeated condition (MS and HS) is not greater than that of the constituents’ non-repeated condition (NU), which does not meet our expectations. We will explain the reason later.

In the EEG results, the results of N400 are consistent with the pattern of behavioral consequences. MS and HS have a significant reduction in amplitude relative to NU, reflecting the repetitive priming of morphemes and semantic priming at the whole word level, which is consistent with the classic N400 effect. What is important is that at these three levels of NU, MS, and HS, RS has a significant reduction in amplitude relative to RD, showing an apparent relation priming effect. The relation priming at the morpheme-repeated level (MS and HS) repeats Ji and Jia et al. [7,8]. A new result we discovered is that the relation priming can also occur independently (NU) without sharing morphemes. It shows that relation can be independently represented and does not have to be bound to morphemes. This aligns with the prediction of the transformational model of relation representation [20]. They suggest that the relation is represented as a unique activation mode triggered by multiple inputs. Initial exposure to a situation will trigger a relation. Then this relation can be applied to a new situation for analogy. In this experiment, regardless of whether there is a repetition of constituents, the activation pattern triggered by the prime can be mapped to the target, thereby facilitating its processing.

The lack of previous findings regarding an independent relation effect can be attributed to several possible factors, which we suppose are as follows: Firstly, the relation effect may be overshadowed by the stronger semantic effect, making it more challenging to detect independently. Secondly, previous studies predominantly relied on traditional reaction-time measures, whereas our experiment utilizes electrophysiological technology, which is more sensitive in capturing cognitive processes. This increased sensitivity may have allowed us to detect the independent relation effect that was previously overlooked. Thirdly, our approach employs a more precise and specific classification of relations, unlike the relatively vague semantic classifications used in prior research. This refined categorization better captures the essence of the relation and enhances our ability to identify its effects. Lastly, due to the emphasis on structural information, including rhyme, in the Chinese language, Chinese individuals, such as our college student participants, are extensively exposed to and experienced in processing structural information from an early age. This familiarity with structural cues may have contributed to our successful detection of the independent relation effect in the N400 window, a novel finding in our study.

The study by Pickering et al. (1998) revealed a notable effect of structure priming in a language production experiment. Since compound words can be seen as the most basic form of language composition, it is reasonable to regard relation priming as a specific type of structural priming. The speaker in their study tends to re-use a specific syntactic structure previously used. For example, suppose the speaker heard a subject-predicate sentence before. In that case, he is more inclined to use subject-predicate sentences rather than prepositional-objective sentences in his following speech, although prepositional-objective sentences can also express the same meaning. When the critical verbs used in the sentence that has been experienced and the sentence that needs to be expressed are the same or have a similar meaning, the tendency to repeat the syntactic structure is particularly strong [21]. This is very similar to relation priming. Only when the prime and target use the same relation and the same morpheme is the relation priming particularly strong. Later, another experiment found that syntactic priming exists independently of the repetition of the concepts (such as verbs) expressed in the sentence [22,23,24]. This is very similar to the discovery process of relation priming. Initially, we thought that relation priming would only occur in the case of morpheme repetition, but now we have found that it could exist independently of morpheme repetition. We think that the essence of syntactic priming and relational priming is the same; both are the embodiment of structure priming at different levels. One is at the sentence level, and the other is at the word level. And they are essentially manifestations of the role of composition rules (structural mapping) in language processing.

Whether muti-morpheme words are processed as a whole or decomposed into smaller units has always been the focus of debate [25,26]. The independent representation of the composition rule (i.e., structure, relation) adds new evidence for the compounds’ decomposition view. However, the materials used in the current experiment are transparent words with relatively low frequency. For compounds with unusually high frequency and compounds that cannot be decomposed literally, whether it is decomposed and processed in this way is worthy of further study in the future.

Based on the ERP data, we examine the time course of relation priming, as shown in Figure 4B,C. During the N200 period, although it did not reach a significant level, the relation was activated to a certain extent at the three levels of NU, MS, and HS, which was manifested in the decrease in the amplitude of RS relative to RD. Seen from the extent of amplitude decline, MS and NU have a certain degree of activation in the early stage of 200ms. The activation increased over time and reached a significant level in the N400 window. At the HS level, there was almost no decrease in the N200 period; that is, there was no activation of relation, but in the N400 period, this activation also reached a significant level, indicating that the relation associated with head activated later relative to MS and NU. In the N200 time window, the relation did not work at the HS level (RS’s amplitude not declined relative to RD). However, its overall activation level is relatively high (amplitude is larger relative to MS and NU), indicating that HS has a relatively high activation level in an earlier period.

Moreover, HS has the shortest response time (MS: 794; HS: 771; NU: 850), indicating that its recognition is the fastest. It shows that although the head is the last morpheme, it plays a more important role in whole word processing than the first morpheme. We guess that the head represents the conceptual category to which the entire word belongs. After it is activated, the approximate meaning of the whole word has been determined. Therefore, in the N200 period, the activation of the head inhibits the activation of the relation associated with it until the later stage of word processing (N400 period), when more precise and detailed information needs to be extracted, and the relation associated with the head is activated. Ji and Gagne’s (2007) results also support our explanation. In their experiment, the modifier or head appeared 350ms before the other constituent to determine the degree to which the relation is related to the two constituents. When the modifier appears 350ms before the head, the relation associated with the head is still active. But, when the head appears 350ms before the modifier, the relation associated with the modifier does not work, indicating that the head plays a more critical role than the modifier in processing Chinese compounds. When the head is pre-activated, the meaning of the entire word has been determined, and the relation associated with the modifier is no longer critical.

Next, we explain the behavioral results. It can be seen from Figure 4A that, for the three morpheme levels, the amplitude reduction in RS relative to RD is the largest at the MS level, followed by the NU, and almost zero at the HS. It indicated that in the modifier-repeated conditions, relation plays the largest role in whole word processing. So the relation priming in the modifier-repeated condition was the first to be discovered by researchers [6,9,10,27]. In combination with the HS level results, we infer that the relation is easier to extract when there is morpheme repetition, regardless of whether it is a modifier or head. Head-repeated condition is an exception, in which relation priming is inhibited by constituent priming, so no effect was manifested. In the no morphemes repeated condition, the relation is more difficult to extract because subjects need to extract the relation of prime without the help of specific concepts and then apply it to target recognition. This process is relatively complicated, so the relation priming shown is smaller than that of MS, and the reaction time is longer.

Therefore, the relation activation pattern of the three levels of morphemes is relation activated at a very early stage (about 200 ms), and morphological processing also occurs in this period (marked by the central-parietal N200). It indicates that the activation of the relation is almost simultaneous with the recognition of the constituent morphemes. In the earlier stage, the degree of activation is relatively small. As word processing progresses, the activation gradually increases. Until the N400 period, the semantic composition begins after the semantic extraction of the constituent morphemes is completed. There are only exceptions in HS. It is equivalent to a one-step success. The activation of constituents (i.e., head) is enough to activate the approximate meaning of the whole word. The relation only started to work when the details needed to be modified later.

## 5. Conclusions

In this study, using Chinese two-morpheme compounds as material, we adopted a priming paradigm to investigate the relation priming of three morphemes level (modifier-repeated, head-repeated, and no repeated morpheme). In addition to the morpheme-repetition condition, we found that the relation priming can also occur without repeated morphemes, indicating that relation can be represented independently and does not have to be bound to a specific morpheme. The results on ERP shows that relation activated as early as about 200ms (N200). With the word processing progress, the activation continues to increase (N400), indicating that relation information plays an increasingly important role in the compound word’s recognition process. First, as contextual information, it helps to determine the meaning of the constituent morphemes. Then after the meaning access of the constituent morphemes, they begin to play a role in the semantic composition process. This study combined behavioral and electrophysiological methods to study the processing process of structural information of compound words comprehensively for the first time, which helps clarify the nature of relation priming. As the first ERP study to discover the independent representation of relation information, it also provides a new perspective for the semantic access process of compound words by depicting the time course of relation information. However, since this study uses Chinese materials and Chinese-speaking subjects, its scope of application is somewhat limited. It is hoped that researchers from other languages can continue to expand this conclusion in the future.

## Figures and Tables

**Table 1:** Example materials.

	Conditions		Examples
Prime	MSRS	Sharing the modifier and the relation with the target	手链 meaning hand chain
	MSRD	Sharing the modifier with but differing in the relation from the target	手背 meaning hand back
	HSRS	Sharing the head and the relation with the target	怀表 meaning pocket watch
	HSRD	Sharing the head with but differing in the relation from the target	秒表 meaning stopwatch
	NURS	Sharing no constituent but having the same relation with the target	颈环 meaning neck ring
	NURD	Sharing neither constituent nor the relation with the target	丝袜 meaning silk stockings
Target			手表 meaning wristwatch

## Data Availability

The data presented in this study are available on request from the first author and the corresponding author.

## References

[1] Murphy G.L. (1990). Noun phrase interpretation and conceptual combination. J. Mem. Lang..

[2] Zhou R.A. (2007). The Syntactic and Semantic Analysis of Noun + Noun Compounds. Ph.D. Thesis.

[3] Raffray C.N., Pickering M.J., Branigan H.P. (2007). Priming the interpretation of noun–noun combinations. J. Mem. Lang..

[4] Gagné C.L., Spalding T.L. (2009). Constituent integration during the processing of compound words: Does it involve the use of relational structures?. J. Mem. Lang..

[5] Chung K.K., Tong X., Liu P.D., McBride-Chang C., Meng X. (2010). The processing of morphological structure information in Chinese coordinative compounds: An event-related potential study. Brain Res..

[6] Gagné C.L., Shoben E.J. (1997). Influence of thematic relations on the comprehension of modifier–noun combinations. J. Exp. Psychol. Learn. Mem. Cogn..

[7] Ji H., Gagné C.L. (2007). Lexical and relational influences on the processing of Chinese modifier-noun compounds. Ment. Lex..

[8] Jia X., Wang S., Zhang B., Zhang J.X. (2013). Electrophysiological evidence for relation information activation in Chinese compound word comprehension. Neuropsychologia.

[9] Gagné C.L. (2001). Relation and lexical priming during the interpretation of noun–noun combinations. J. Exp. Psychol. Learn. Mem. Cogn..

[10] Gagné C.L., Shoben E.J. (2002). Priming relations in ambiguous noun-noun combinations. Mem. Cogn..

[11] Gagné C.L., Spalding T.L., Ji H. (2005). Re-examining evidence for the use of independent relational representations during conceptual combination. J. Mem. Lang..

[12] Estes Z., Jones L.L. (2006). Priming via relational similarity: A copper horse is faster when seen through a glass eye. J. Mem. Lang..

[13] Zhang J.X. (2011). The meaning-spelling theory of Chinese character: The new definition of the nature of Chinese characters from a psychological perspective. J. South China Norm. Univ. Soc. Sci. Ed..

[14] Zhang J.X., Fang Z., Du Y., Kong L., Zhang Q., Xing Q. (2012). Centro-parietal N200: An event-related potential component specific to Chinese visual word recognition. Chin. Sci. Bull..

[15] Manouilidou C., Ralli A., Kordouli K. (2012). Coordinative compounds in Greek: Lexical access and representation. Lingue Ling..

[16] Cai Q., Brysbaert M. (2010). SUBTLEX-CH: Chinese word and character frequencies based on film subtitles. PLoS ONE.

[17] Baayen R.H., Davidson D.J., Bates D.M. (2008). Mixed-effects modeling with crossed random effects for subjects and items. J. Mem. Lang..

[18] Kuznetsova A., Brockhoff P.B., Christensen R.H. (2017). lmerTest package: Tests in linear mixed effects models. J. Stat. Softw..

[19] (2013). R: A language and Environment for Statistical Computing.

[20] Leech R., Mareschal D., Cooper R.P. (2008). Analogy as relational priming: A developmental and computational perspective on the origins of a complex cognitive skill. Behav. Brain Sci..

[21] Pickering M.J., Branigan H.P. (1998). The representation of verbs: Evidence from syntactic priming in language production. J. Mem. Lang..

[22] Cai Z.G., Pickering M.J., Branigan H.P. (2012). Mapping concepts to syntax: Evidence from structural priming in Mandarin Chinese. J. Mem. Lang..

[23] Huang J., Pickering M.J., Yang J., Wang S., Branigan H.P. (2016). The independence of syntactic processing in Mandarin: Evidence from structural priming. J. Mem. Lang..

[24] Yu Z., Zhang Q. (2020). The influence of syntactic structure and verb repetition on the syntactic priming effect of spoken Chinese sentences. Chin. J. Psychol..

[25] Badecker W. (2007). Processing compound words: An introduction to the issues. Brain Lang..

[26] Taft M., Forster K.I. (1975). Lexical storage and retrieval of prefixed words. J. Verbal Learn. Verbal Behav..

[27] Gagné C.L. (2000). Relation-based combinations versus property-based combinations: A test of the CARIN theory and the dual-process theory of conceptual combination. J. Mem. Lang..

